# Common Polymorphism in the *LRP5* Gene May Increase the Risk of Bone Fracture and Osteoporosis

**DOI:** 10.1155/2014/290531

**Published:** 2014-12-14

**Authors:** Guang-Yue Xu, Yong Qiu, Hai-Jun Mao

**Affiliations:** Department of Orthopaedics, Drum Tower Hospital, Medical School of Nanjing University, Zhongshan Road No. 321, Nanjing 210008, China

## Abstract

The low-density lipoprotein receptor-related protein 5 gene (*LRP5*) was identified to be linked to the variation in bone mineral density and types of bone diseases. The present study was aimed at examining the association of *LRP5* rs3736228 C>T gene with bone fracture and osteoporosis by meta-analysis. A systematic electronic search of literature was conducted to identify all published studies in English or Chinese on the association of the *LRP5* gene with bone fracture and osteoporosis risks. All analyses were calculated using the Version 12.0 STATA software. Odds ratios (ORs) and their corresponding 95% confidence interval (95% CI) were calculated. An updated meta-analysis was currently performed, including seven independent case-control studies. Results identified that carriers of rs3736228 C>T variant in the *LRP5* gene were associated with an increased risk of developing osteoporosis and fractures under 4 genetic models but not under the dominant model (OR = 1.19, 95% CI = 0.97~1.46, and *P* = 0.103). Ethnicity-subgroup analysis implied that *LRP5* rs3736228 C>T mutation was more likely to develop osteoporosis and fractures among Asians and Caucasians in majority of subgroups. These results suggest that there is a modest effect of the *LRP5* rs3736228 C>T on the increased susceptibility of bone fracture and osteoporosis.

## 1. Introduction

Bone fracture, mainly caused by decrease in bone strength associated with bone loss, has contributed to an increase in disability, morbidity, death, and health expenses [1, 2]. According to a recent estimation, 200 in every 1000 people would suffer a bone fracture during their whole lifetime, which imposes huge burden on public health services worldwide [1, 3]. It has been reported that a wide range of risk factors, such as HIV infection, obesity, fibrous dysplasia of bone, age, and gender, together with genetic factors, have played pivotal roles in the pathogenesis of bone fractures [2, 4, 5]. In general, osteoporosis, characterized by a progressive degeneration of bone tissues and a low bone mineral density, is widely accepted as a secret as well as systemic skeletal disease, without being detected by a majority of its infected persons [6]. Previous researches have showed that osteoporosis would increase bone fragility subsequently and is susceptible to fracture; meanwhile, it has been demonstrated to affect over 75 million people all over the world [6, 7]. As a multifactorial disease, the etiology of osteoporosis is complicated, mainly attributed to interactions between family genetic history and environmental risk factors [8]. Multiple environmental factors, including physical activity, dietary, age, cigarette smoking, malabsorption, and nutritional status have had huge effects on the development of osteoporosis [9, 10]. Recently, many studies have emphasized exploring the relationships of clinical biomarkers with bone fracture and osteoporosis, and lipoprotein receptor-related protein 5 (LRP5), whose mutations would reduce bone mineral density, is thought to be corrected with the susceptibility to osteoporosis [11, 12].

Lipoprotein receptor-related protein 5 (LRP5), as a member of the low-density lipoprotein receptor family, is a single-pass plasma membrane protein secreted in many tissues and cells, such as breast tissues, bone tissues, endothelial cells, and stem cells [13, 14]. Human* LRP5* gene is located on chromosome 11q13.4 and consists of 22 introns and 23 exons, spanning approximately 160 kb [15]. It has been revealed that LRP5 has a huge effect on Wnt signaling pathway, which is closely related to the regulation of osteoblasts growth and differentiation by controlling bone density as well as bone metabolism [16]. In addition, LRP5 also plays a pivotal role in blood lipid metabolism and blood glucose, resulting in prevention of decreased bone formation; thus LRP5 is essential for bone development and health [17, 18]. However, recent researches have showed that loss-of-function mutations of the* LRP5* gene contribute to subsequent reduction of bone mineral density (BMD), indicating a dominant negative effect on bone mass, which would lead to various bone diseases [17, 19]. A few common polymorphisms of the* LRP5* gene have been detected in correlation with bone phenotypes, including fracture risk and BMD, among which a coding single nucleotide polymorphism (SNP) of the* LRP5* gene, rs3736228 (A1330V), is thought to have a particular susceptibility to osteoporosis [11]. The polymorphism rs3736228, located in exon 18, would restrict the expression of Tph1 in the duodenum enterochromaffin cells, which adjusts bone formation, as well as BMD, and finally lead to osteoporosis or even bone fracture [20, 21]. Therefore, it can be speculated that SNP rs3736228 C>T of the* LRP5* gene could be regarded as a useful genetic biomarker for the prediction of osteoporosis and bone fracture [22]. Nowadays, there was no meta-analysis focused on the relationships between polymorphism rs3736228 C>T in the* LRP5* gene and the risk of bone fracture, as well as osteoporosis. This study aimed to give an overall view of this subject and further evaluate its role as a biomarker in predicting the pathogenesis of osteoporosis.

## 2. Materials and Methods

### 2.1. Literature Search and Data Sources

Potential relevant studies were identified by a comprehensive literature search in April 30, 2014, which included the following computerized bibliographic databases: MEDLINE (1966~2014), Science Citation Index (1945~2014), Cochrane Library (Oxford, UK, Issue 12, 2014), PubMed (1966~2014), Embase (1974~2014), CINAHL (1982~2014), and Current Contents Index (1995~2014). In addition, Chinese Biomedical (1978~2014), Chinese Journal Full-Text (1980~2014), and Weipu Journal (1989~2014) were also used to identify Chinese articles. We used medical subject headings and free language terms with a highly sensitive search strategy, the search terms were as follows: “Fractures, Bone” or “Broken Bones” or “Fractures” or “Fracture” or “Broken Bone” or “Bone Fractures” or “Bone Fracture” and “OP, Postmenopausal” or “OP” or “Juvenile OP” or “OP” or “Osteoporoses” or “Age-Related Bone Loss” or “Age-Related OP” or “Age-Related OP” and “LRP5 protein, human” or “LRP5” or “low density lipoprotein receptor-related protein 5” or “LDL receptor-related protein 5.” No restrictions were made with respect to language, country, or data collection. Manual searches were also used to identify other potential articles. Further trials were sought from reference lists in the relevant papers to find additional works which failed to be captured by electronic or manual searches.

### 2.2. Inclusion and Exclusion Criteria

To be included in the systematic review, retrieved studies had to be assessed with two observers (Jia-Li Liu and Yong-Bing Xiang) for their suitability for meeting the following criteria: (1) case-control studies published on peer-reviewed journals; (2) only those studies examining the associations between SNPs in* LRP5* (rs3736228) polymorphism and susceptibility to osteoporosis and fracture were incorporated into the meta-analysis; (3) all subjects underwent diagnostic evaluations and satisfied the clinical diagnosis of osteoporosis or fracture confirmed by the report of the World Health Organization Task-Force for Osteoporosis [23, 24]; (4) the article must provide original data and contain sufficient information on the genotype frequencies of the rs3736228 C>T polymorphism within the* LRP5* gene; (5) distributions of genotype frequencies in* LRP5* (rs3736228 C>T) were within the range of Hardy-Weinberg equilibrium (HWE) in the controls; (6) once studies provided overlapping data, we would choose the study that had the largest sample size. The major exclusion criteria in this meta-analysis were as follows: (1) some publication types presenting nonoriginal data, such as letters, editorials, abstracts, reviews, meta-analysis opinion papers or proceedings; (2) unpublished sources of data; (3) duplicated publications or studies without extractable, numerical data; (4) subgroup analysis of the included trials. Based on these inclusion/exclusion criteria, the title and abstract of all the retrieved articles were evaluated for relevance, and then the full texts of the selected articles were reviewed, followed by a decision on their eligibility for inclusion in this systematic review.

### 2.3. Study Quality and Data Extraction

In order to ensure consistency in reviewing and reporting results, two reviewers independently assessed the methodological quality of the included trials using the Newcastle-Ottawa Scale (NOS) criteria with study design, content, and ease-of-use in the explanation of results or the meta-analysis for assessing the quality [25]. The three broad perspectives were judged: (1) subject selection: 0~4; (2) subject comparability: 0~2; (3) clinical outcome: 0~3. The NOS scores ranged from 0 to 9; a study was in a good quality for the evidence of a score ≥7.

Each of the two reviewers assessed the studies independently based on the inclusion/exclusion criteria mentioned before to the methods section. We used a standardized data form in duplicate to collect the following descriptive information: surname and initials of the first author, the year of publication or submission, journal, source country, racial descent of study population, language of publication, study design, number of cases and controls, source of controls, demographic variables of the subjects, SNP information, detection method of genotypes, genotype frequencies, allele frequencies, HWE test and confirmation of diagnosis, and so forth. Disagreement on the inclusion of a single study was settled by discussion, or a third investigator was consulted.

### 2.4. Statistical Analysis

We calculated the odds ratios (ORs) and their corresponding 95% confidence interval (CI), for the purpose of evaluating the relationship of the SNP in* LRP5* (rs3736228 C>T) with bone fracture and osteoporosis. A 95% confidence interval (95% CI) was calculated for the summary OR by the use of *Z*  test. The pooled ORs were carried out for the comparison in allele model (W allele versus M allele), dominant model (WW + WM versus MM), recessive model (WW versus WM + MM), homozygous model (WW versus MM), and heterozygous model (WW versus WM), respectively. Also, in order to explore for heterogeneity other than threshold effect, a test for heterogeneity between trials included for each comparison was performed by the use of the Cochran's *Q*-statistic and *I* ^2^ tests [26]. If the *Q*-test showed evidence of a *P* < 0.05 or *I* ^2^ test exhibited > 50%, indicating maximal heterogeneity among the included studies, we did metaregression analysis to explore sources of heterogeneity with a random-effects model by relating study level covariates to diagnostic OR, and otherwise ORs were pooled according to the fixed-effects model [27, 28]. When a substantial heterogeneity was found, the differences in genotype/allele frequencies in* LRP5* (rs3736228 C>T) (and 95% CI) were evaluated for subgroups of different explanatory variables. Additionally, in order to evaluate the impact of single studies on the overall estimate, a one-way sensitivity analysis was employed to ensure that no single study was completely responsible for the overall results. Further, Egger's linear regression test with visual inspection of the funnel plot was applied to detect the potential publication bias [29, 30]. Statistical analyses were conducted with the STATA statistical software (Version 12.0, Stata Corporation, College Station, TX, USA).

## 3. Results

### 3.1. Description of Included Studies

**Figure 1:**
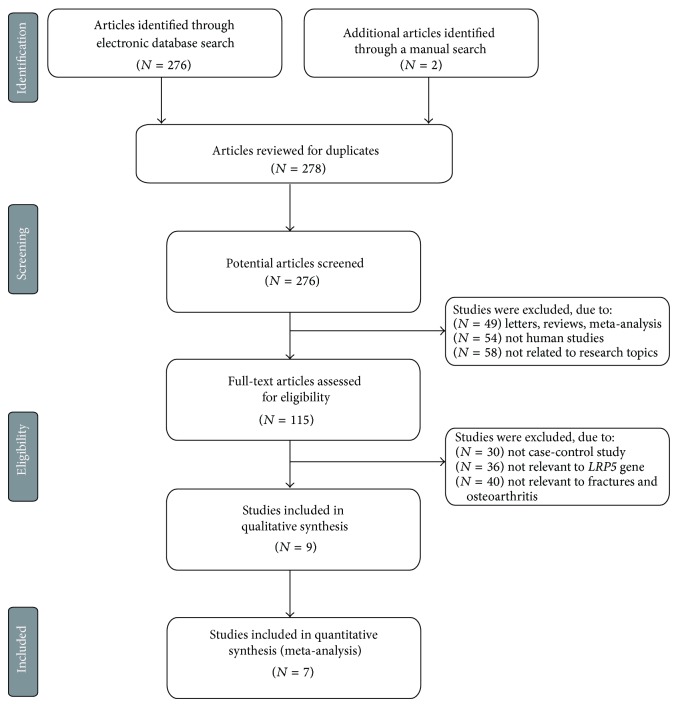
Flow chart shows study selection procedure. Seven case-control studies were included in this meta-analysis.

The combined electronic and manual search initially resulted in 278 potentially eligible articles. After the exception of 2 duplicated studies, 276 retrieved studies were screened by title and abstract for relevance; subsequently, 161 irrelevant articles were excluded. Then, we systematically reviewed the remaining 115 articles qualified for full-text reading, and 106 articles were deemed unsuitable and were therefore excluded. Thus, 9 articles were identified to be included in quantitative analysis. In addition, another 2 studies were excluded due to lack of data integrity after a more careful assessment of the remaining articles. Finally, 7 studies composed of 2,772 subjects including 907 patients and 1,865 control subjects were incorporated into this meta-analysis [16, 22, 31–35].  Figure 1 presented the progress of study selection and the main reason for exclusion. All the enrolled papers showed moderate-high quality.

From the 7 included studies, 2 studies focused on the genotype/allele frequencies of* LRP5* (rs3736228 C>T) in fracture patients and the controls; the other 5 studies were concerned about the genotype/allele frequencies of* LRP5* (rs3736228 C>T) in osteoporosis patients and the controls. Additionally, of the 7 studies included in the analysis, 3 were performed in Caucasians and the other 4 were in Asians. The controls were drawn from population-based sources and hospital-based sources. With respect to the genotyping methods, two studies were performed with non-TaqMan assay (direct sequencing and pyrosequencing), and the other five studies were conducted with TaqMan assay. The information of the SNPs (rs3736228 C>T) in the* LRP5* gene was included to evaluate the association between polymorphic variants of the* LRP5* gene and the risk of osteoporosis and fractures.  Table 1 showed the baseline characteristics and the genotype/frequencies of rs3736228 C>T SNP in the individual studies.

### 3.2. Quantitative Data Synthesis

**Figure 2:**
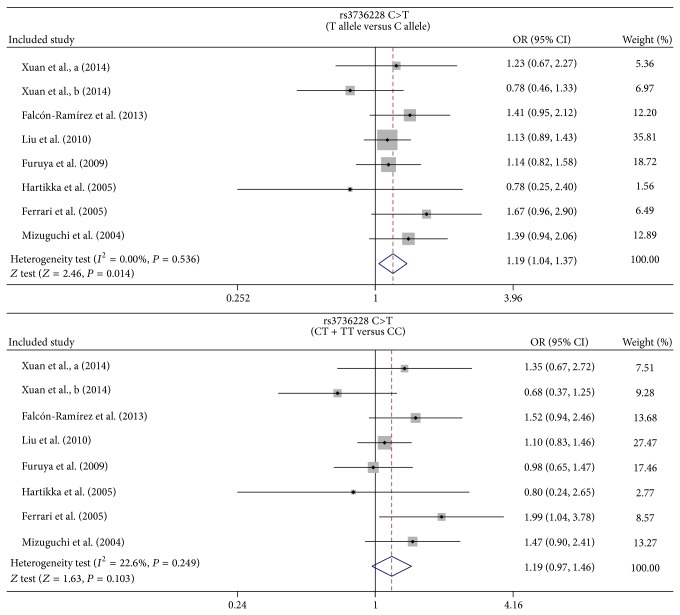
Forest plots for the relationships between* LRP5* rs3736228 C>T polymorphism and the development of bone fracture and osteoporosis under the allele and dominant models.

In this meta-analysis, one SNP within the* LRP5* gene was identified, and the association between the allelic and genotypic frequencies of* LRP5* rs3736228 C>T and the risk of osteoporosis and fractures were investigated. Results in this meta-analysis demonstrated that the carriers of the rs3736228 C>T polymorphism in the* LRP5* gene were associated with an increased risk of developing osteoporosis and fractures in the allele model, whereas the dominant model of* LRP5* rs3736228 C>T showed no statistically significant differences between the cases and controls derived from the 7 included studies (OR = 1.19, 95% CI = 0.97~1.46, and *P* = 0.103) (Figure 2).

**Figure 3:**
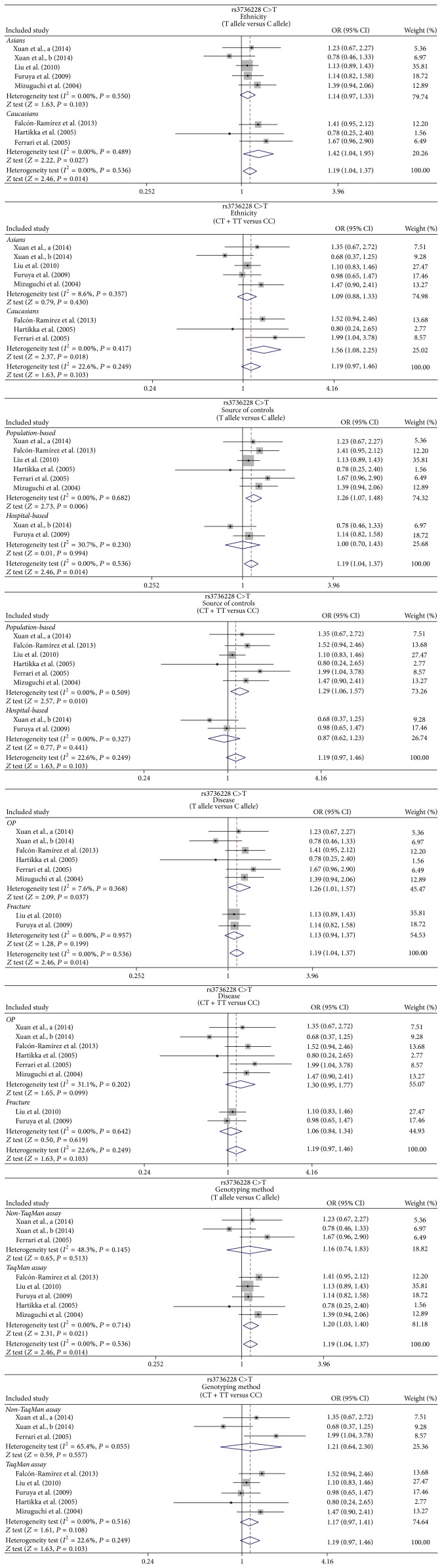
Subgroup analysis for the relationships between* LRP5* rs3736228 C>T polymorphism and the development of bone fracture and osteoporosis under the allele and dominant models.

In the ethnicity-stratified subgroups, we found that the* LRP5* rs3736228 C>T polymorphism-containing populations were more likely to develop osteoporosis and fractures in the Caucasians under both the allele and dominant models (T allele versus C: OR = 1.42, 95% CI = 1.04~1.95, and *P* = 0.027; CT + TT versus CC: OR = 1.56, 95% CI = 1.08~2.25, and *P* = 0.018), yet similar results were not observed among Asians (*P* > 0.05) (as shown in Figure 3). When stratified by the source of controls, the results yielded increased risk of osteoporosis and fractures in rs3736228 carriers in the allele, dominant, and homozygous models in the population-based subgroup (T allele versus C: OR = 1.26, 95% CI = 1.07~1.48, and *P* = 0.006; CT + TT versus CC: OR = 1.29, 95% CI = 1.06~1.57, and *P* = 0.010; TT versus CC: OR = 1.64, 95% CI = 1.02~2.65, and *P* = 0.042, resp.) and in recessive model, homozygous model, and heterozygous model in the hospital-based subgroups (TT versus CC + CT: OR = 2.24, 95% CI = 1.12~4.46, and *P* = 0.022; TT versus CC: OR = 2.06, 95% CI = 1.02~4.17, and *P* = 0.045; TT versus CT: OR = 2.56, 95% CI = 1.23~5.31, and *P* = 0.012, resp.) (Table 2). For the disease based-subgroups, subjects with rs3736228 polymorphism were more likely to develop osteoporosis in the allele model (OR = 1.26, 95% CI = 1.01~1.57, and *P* = 0.037) and fractures in the in recessive, homozygous, and heterozygous models (all *P* < 0.05). When concerned about the genotype method-stratified subgroups, it has been revealed that the rs3736228 C>T polymorphism carriers in the allele, recessive, homozygous, and heterozygous models were related to significantly higher osteoporosis and fractures susceptibility in the TaqMan assay subgroup (all *P* < 0.05), yet it showed none significant association regarding the rs3736228 C>T polymorphism and higher risks of osteoporosis and fractures in the non-TaqMan assay subgroup under 5 genetic models (all *P* > 0.05) (Table 2, Figure 3). However, neither univariate nor multivariate metaregression analyses showed any evidence of the potential source of heterogeneity in ethnicity, source of controls, and disease or genotyping method (all *P* > 0.05) (Table 3).

**Figure 4:**
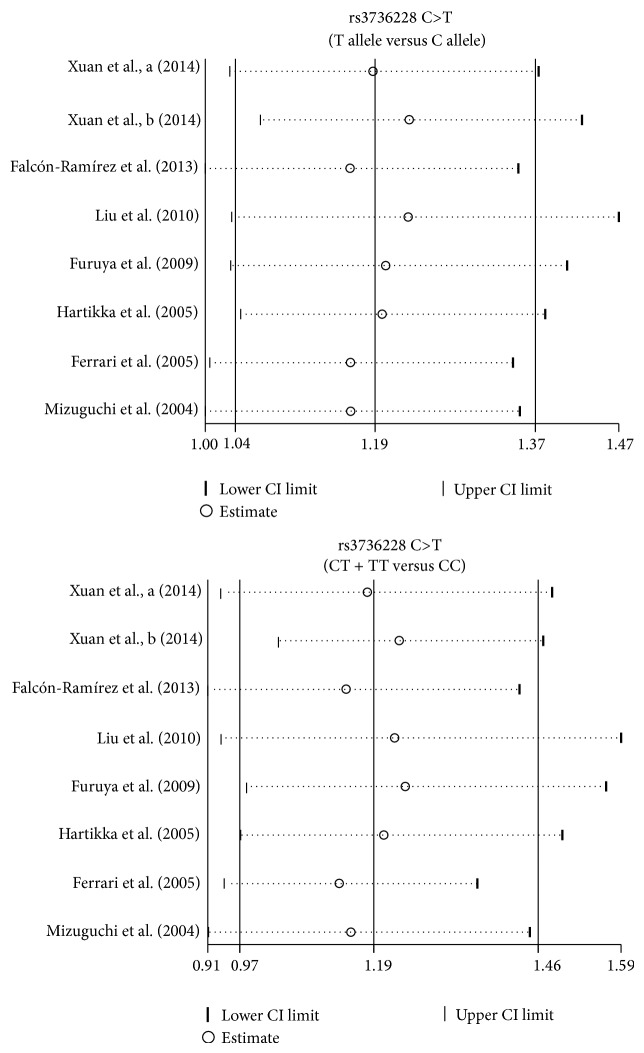
Sensitivity analysis for the relationships between* LRP5* rs3736228 C>T polymorphism and the development of bone fracture and osteoporosis under the allele and dominant models.

**Figure 5:**
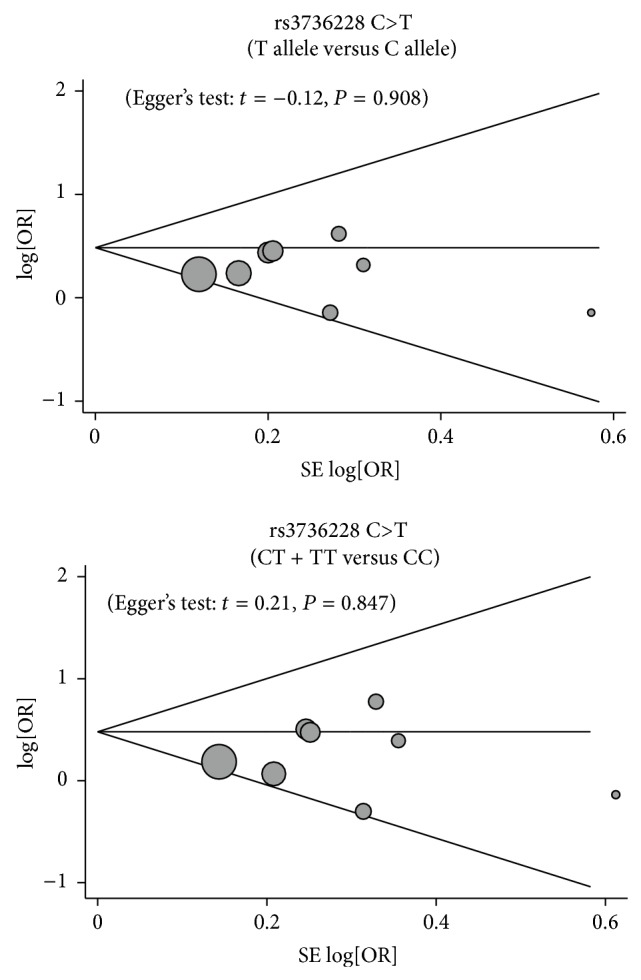
Funnel plot of publication biases for the relationships between* LRP5* rs3736228 C>T polymorphism and the development of bone fracture and osteoporosis under the allele and dominant models.

We further conducted sensitivity analyses to determine whether review conclusions were affected by the choice of single study; the finding suggested that no single study had the effect on the pooled ORs in the current meta-analysis (Figure 4). Finally, the Egger's test applied to the detection of publication bias presented no evidence of asymmetrical distribution in the funnel plot, suggesting that publication bias was not detected in the rs3736228 C>T allele model (*t* = −0.12, *P* = 0.908) and rs3736228 C>T dominant model (*t* = 0.21, *P* = 0.847) in systematic reviews (Figure 5).

## 4. Discussion

The present meta-analysis aggregated large-scale evidence from relevant studies in an attempt to determine whether rs3736228 C>T polymorphism in the* LRP5* gene was related with susceptibility to bone fracture and osteoporosis. The main findings of our statistical analysis indicated that* LRP5* rs3736228 C>T polymorphism might be connected with the pathogenesis of bone fracture and osteoporosis, demonstrating that this polymorphism may be implicated in the development of bone fracture and osteoporosis, which is manifested by reduced bone strength and increased susceptibility to fracture. However, the mechanism underlying the pathogenesis of bone fracture and osteoporosis has remained poorly understood. As a critical member of the low-density lipoprotein (LDL) receptor family, LRP5 may bind and internalize ligands in the process of receptor-mediated endocytosis and also play a crucial role in skeletal homeostasis [32, 36]. In general, LRP5 acts as a wingless (Wnt) coreceptor for Frizzled (Fz) receptors family that is responsible for the activation of the Wnt/*β*-catenin canonical pathway [13]. It has been demonstrated that the Wnt signaling pathway has an important role in the formation of bone and the pathogenesis of osteoporosis, and that LRP5 signaling is necessary for normal morphology, developmental processes, and bone health [37]. Furthermore, LRP5 is capable of regulating the growth and differentiation of osteoblasts [38]. Recently, genetic variants in the* LRP5* gene have been reported to be associated with the risk of bone fracture and osteoporosis [31, 39]. Actually,* LRP5* genetic polymorphisms might cause loss of function of LRP5, decrease the signaling activity of the canonical Wnt signaling pathway, and lead to reduced bone formation, thereby conducing to the development of bone fracture and osteoporosis [39, 40]. Consistent with our findings, van Meurs et al. have suggested in their study that common genetic variants in the* LRP5* gene might be consistently linked to bone mineral density and the risk of bone fracture across different white populations [14]. Ferrari et al. also found that* LRP5* genetic polymorphisms seem to be possible genetic determinants for susceptibility to idiopathic osteoporosis in males [31].

Results of ethnicity-stratified analysis revealed that rs3736228 C>T variant might be connected with bone fracture and osteoporosis risks among Caucasians but not among Asians. Type of disease-stratified analysis indicated that rs3736228 C>T mutation might be related to the development of osteoporosis, but this polymorphism may not be a predictive factor for the etiology of bone fracture. In summary, the discovery of the represented meta-analysis was in conformity with previous studies that* LRP5* rs3736228 C>T polymorphism might be closely implicated in the pathogenesis of bone fracture and osteoporosis, implying that this polymorphism may be a helpful biomarker in predicting the occurrence of bone fracture and osteoporosis.

Indeed, some advantages could be highlighted in this meta-analysis. One of the major superiorities may be that the present research shed lights on the relation of genetic polymorphisms in* LRP5*, especially the rs3736228 C>T variant, and the increased susceptibility to bone fracture and osteoporosis, comprehensively and systematically. Additionally, all included literatures had acceptable quality scores (quality scores were higher than seven). However, some limitations of this meta-analysis should also be acknowledged when interpreting the results. Firstly, the current analysis was only limited to one single SNP (rs3736228 C>T) that is being widely discussed in various researches, while other SNPs have also been researched to be related to bone fracture and osteoporosis risk. One of the major concerns may be the bias due to selective publication and language bias derived from the fact that the screened references of papers published in languages other than English and Chinese were not included. Secondly, the crude division criteria of ethnic groups into “Caucasians,” and “Asians” promoting the study are prone to bias. All studies were performed in Asians and Caucasians; to capture the full range of possible ethnic differences in* LRP5* rs3736228 C>T polymorphisms, further studies are needed in other ethnic groups, such as among Africans. Thus, deeper investigation from different populations is warranted to clarify the present results. Another important concern should take into consideration that different diseases have different risk factors and diverse sensitivities to them. In particular, we did not evaluate family history and clinical implication of bone fracture or osteoporosis in our study since we did not collected those information at baseline. Finally, the present sample size did limit the power to identify* LRP5* rs3736228 polymorphism with a small influence on bone fracture and osteoporosis.

In summary, this meta-analysis suggests that rs3736228 C>T variant in the* LRP5* gene may increase the risk of bone fracture and osteoporosis. SNP in the* LRP5* gene may considerably act as a potential candidate of biomarker for bone fracture and osteoporosis screening, diagnosis, and future treatment. To certain the current results, updated well-designed researches with larger sample size, in diverse ethnic populations particularly, are required in the future.

## Figures and Tables

**Table 1:** Baseline characteristics and methodological quality of all included studies.

First author	Year	Ethnicity	Disease	Sample size		Gender (M/F)		Age (years)		Genotyping methods	NOS score
				Case	Control	Case	Control	Case	Control		
Xuan [33]	2014	Asians	OP	90	78	0/90	0/78	60.0 ± 3.0	60.5 ± 2.7	PCR-RFLP	8
				90	96	0/90	0/78	60.0 ± 3.0	60.4 ± 3.2	PCR-RFLP	
Falcón-Ramírez [22]	2013	Caucasians	OP	100	217	0/100	0/217	64.7 ± 10.5	57.4 ± 12.0	TaqMan assay	8
Liu [34]	2010	Asians	Fracture	284	728	0/284	0/728	60.1 ± 10.5	54.9 ± 11.2	TaqMan assay	8
Furuya [16]	2009	Asians	Fracture	119	441	0/95	0/441	—	—	TaqMan assay	8
Hartikka [35]	2005	Caucasians	OP	20	88	11/9	—	10.0 (4.0~16.0)	—	TaqMan assay	7
Ferrari [31]	2005	Caucasians	OP	78	86	78/0	86/0	50.7 ± 9.9	49.8 ± 16.1	Pyrosequencing	8
Mizuguchi [32]	2004	Asians	OP	126	131	0/126	0/131	—	—	TaqMan assay	7

M: male, F: female, PCR-RFLP: polymerase chain reaction-restriction fragment length polymorphism, NOS: Newcastle-Ottawa Scale.

**Table 2:** Meta-analysis of the relationships of *LRP5* rs3736228 polymorphism with bone fracture and osteoporosis.

Subgroup analysis	T allele versus C (allele model)			CT + TT versus CC (dominant model)			TT versus CC + CT (recessive model)			TT versus CC (homozygous model)			TT versus CT (heterozygous model)		
	OR	95% CI	*P*	OR	95% CI	*P*	OR	95% CI	*P*	OR	95% CI	*P*	OR	95% CI	*P*
Overall	1.19	1.04–1.37	0.014	1.19	0.97–1.46	0.103	1.72	1.16–2.53	0.007	1.76	1.19–2.62	0.005	1.62	1.08–2.44	0.020
Ethnicity															
Asian	1.14	0.97–1.33	0.103	1.09	0.88–1.33	0.430	1.75	1.15–2.66	0.009	1.76	1.15–2.70	0.009	1.72	1.11–2.67	0.016
Caucasian	1.42	1.04–1.37	0.027	1.56	1.08–2.25	0.018	1.51	0.53–4.32	0.438	1.77	0.61–5.10	0.290	1.15	0.39–3.38	0.795
Source of controls															
PB	1.26	1.01–1.57	0.037	1.29	1.06–1.57	0.010	1.52	0.95–2.43	0.083	1.64	1.02–2.65	0.042	1.32	0.81–2.16	0.263
HB	1.13	0.94–1.37	0.199	0.87	0.62–1.23	0.441	2.24	1.12–4.46	0.022	2.06	1.02–4.17	0.045	2.56	1.23–5.31	0.012
Disease															
OP	1.26	1.01–1.57	0.037	1.30	0.95–1.77	0.099	1.47	0.79–2.73	0.219	1.63	0.87–3.05	0.124	1.24	0.65–2.35	0.508
Fracture	1.13	0.94–1.37	0.199	1.06	0.84–1.34	0.619	1.90	1.15–3.14	0.012	1.86	1.11–3.10	0.018	1.95	1.15–3.29	0.013
Genotyping method															
Non-TaqMan assay	1.16	0.74–1.83	0.513	1.21	0.64–2.30	0.557	1.13	0.40–3.19	0.813	1.20	0.42–3.39	0.735	1.00	0.34–2.92	0.997
TaqMan assay	1.20	1.03–1.40	0.021	1.17	0.97–1.41	0.108	1.84	1.21–2.80	0.005	1.88	1.23–2.89	0.004	1.76	1.13–2.73	0.012

OR: odds ratio and 95% CI: 95% confidence interval.

**Table 3:** Univariate and multivariate metaregression analyses of potential source of heterogeneity.

Heterogeneity factors	Coefficient	SE	*Z*	*P*	95% CI	
					LL	UL
Publication year						
Univariate	−0.025	0.023	−1.06	0.288	−0.070	0.021
Multivariate	−0.022	0.025	−0.91	0.361	−0.071	0.026
Ethnicity						
Univariate	0.223	0.179	1.25	0.213	−0.127	0.573
Multivariate	0.194	0.224	0.86	0.388	−0.246	0.633
Source of controls						
Univariate	−0.199	0.164	−1.21	0.225	−0.521	0.123
Multivariate	−0.116	0.180	−0.65	0.518	−0.468	0.236
Disease						
Univariate	−0.113	0.144	−0.79	0.432	−0.396	0.169
Multivariate	−0.006	0.213	−0.03	0.977	−0.425	0.412
Genotyping method						
Univariate	−0.199	0.164	−1.21	0.225	−0.521	0.123
Multivariate	−0.023	0.237	−0.10	0.923	−0.487	0.441

SE: standard error, 95% CI: 95% confidence interval, UL: upper limit, and LL: lower limit.

## References

[1] Wang X.-F., Seeman E. (2012). Epidemiology and structural basis of racial differences in fragility fractures in Chinese and Caucasians. *Osteoporosis International*.

[2] Pressley J. C., Kendig T. D., Frencher S. K., Barlow B., Quitel L., Waqar F. (2011). Epidemiology of bone fracture across the age span in blacks and whites. *The Journal of trauma*.

[3] Banu J. (2013). Causes, consequences, and treatment of osteoporosis in men. *Drug Design, Development and Therapy*.

[4] Ofotokun I., Weitzmann M. N. (2010). HIV-1 infection and antiretroviral therapies: risk factors for osteoporosis and bone fracture. *Current Opinion in Endocrinology, Diabetes and Obesity*.

[5] Kim J.-E., Hsieh M.-H., Soni B. K., Zayzafoon M., Allison D. B. (2013). Childhood obesity as a risk factor for bone fracture: a mechanistic study. *Obesity*.

[6] Hadji P., Klein S., Gothe H. (2013). The epidemiology of osteoporosis—Bone Evaluation Study (BEST): an analysis of routine health insurance data. *Deutsches Ärzteblatt International*.

[7] Lewiecki E. M., Bilezikian J. P., Bonewald L., Compston J. E., Heaney R. P., Kiel D. P., Miller P. D., Schousboe J. T. (2014). Osteoporosis update: proceedings of the 2013 santa fe bone symposium. *Journal of Clinical Densitometry*.

[8] Raje M., Botre C., Ashma R. (2013). Genetic epidemiology of osteoporosis across four microsatellite markers near the VDR gene. *International Journal of Molecular Epidemiology and Genetics*.

[9] Lehouck A., Boonen S., Decramer M., Janssens W. (2011). COPD, bone metabolism, and osteoporosis. *Chest*.

[10] Cummins N. M., Poku E. K., Towler M. R., O'Driscoll O. M., Ralston S. H. (2011). Clinical risk factors for osteoporosis in Ireland and the UK: a comparison of FRAX and QFractureScores. *Calcified Tissue International*.

[11] Kruk M., Ralston S. H., Albagha O. M. E. (2009). LRP5 polymorphisms and response to risedronate treatment in osteoporotic men. *Calcified Tissue International*.

[12] Saarinen A., Saukkonen T., Kivelä T., Lahtinen U., Laine C., Somer M., Toiviainen-Salo S., Cole W. G., Lehesjoki A.-E., Mäkitie O. (2010). Low density lipoprotein receptor-related protein 5 (LRP5) mutations and osteoporosis, impaired glucose metabolism and hypercholesterolaemia. *Clinical Endocrinology*.

[13] Marques-Pinheiro A., Levasseur R., Cormier C., Bonneau J., Boileau C., Varret M., Abifadel M., Allanore Y. (2010). Novel LRP5 gene mutation in a patient with osteoporosis-pseudoglioma syndrome. *Joint Bone Spine*.

[14] van Meurs J. B. J., Trikalinos T. A., Ralston S. H., Balcells S., Brandi M. L., Brixen K., Kiel D. P., Langdahl B. L., Lips P., Ljunggren Ö., Lorenc R., Obermayer-Pietsch B., Ohlsson C., Pettersson U., Reid D. M., Rousseau F., Scollen S., Van Hul W., Agueda L., Åkesson K., Benevolenskaya L. I., Ferrari S. L., Hallmans G., Hofman A., Husted L. B., Kruk M., Kaptoge S., Karasik D., Karlsson M. K., Lorentzon M., Masi L., McGuigan F. E. A., Mellström D., Mosekilde L., Nogues X., Pols H. A. P., Reeve J., Renner W., Rivadeneira F., Van Schoor N. M., Weber K., Ioannidis J. P. A., Uitterlinden A. G. (2008). Large-scale analysis of association between LRP5 and LRP6 variants and osteoporosis. *Journal of the American Medical Association*.

[15] Smith A. J. P., Gidley J., Sandy J. R., Perry M. J., Kirwan J. R., Spector T. D., Doherty M., Bidwell J. L., Mansell J. P. (2005). Haplotypes of the low-density lipoprotein receptor-related protein 5 (LRP5) gene: are they a risk factor in osteoarthritis?. *Osteoarthritis and Cartilage*.

[16] Furuya T., Urano T., Ikari K., Kotake S., Inoue S., Hara M., Momohara S., Kamatani N., Yamanaka H. (2009). A1330V polymorphism of low-density lipoprotein receptor-related protein 5 gene and self-reported incident fractures in Japanese female patients with rheumatoid arthritis. *Modern Rheumatology*.

[17] Agueda L., Velázquez-Cruz R., Urreizti R., Yoskovitz G., Sarriõn P., Jurado S., Güerri R., Garcia-Giralt N., Nogués X., Mellibovsky L., Díez-Pérez A., Marie P. J., Balcells S., Grinberg D. (2011). Functional relevance of the BMD-associated polymorphism rs312009: novel involvement of RUNX2 in LRP5 transcriptional regulation. *Journal of Bone and Mineral Research*.

[18] Laine C. M., Chung B. D., Susic M., Prescott T., Semler O., Fiskerstrand T., D'Eufemia P., Castori M., Pekkinen M., Sochett E., Cole W. G., Netzer C., Mäkitie O. (2011). Novel mutations affecting LRP5 splicing in patients with osteoporosis-pseudoglioma syndrome (OPPG). *European Journal of Human Genetics*.

[19] Goltzman D. (2011). LRP5, serotonin, and bone: complexity, contradictions, and conundrums. *Journal of Bone and Mineral Research*.

[20] Tran B. N. H., Nguyen N. D., Eisman J. A., Nguyen T. V. (2008). Association between LRP5 polymorphism and bone mineral density: a Bayesian meta-analysis. *BMC Medical Genetics*.

[21] Funakoshi Y., Omori H., Yada H., Katoh T. (2010). Relationship between changes of bone mineral density over seven years and A1330V polymorphism of the low-density lipoprotein receptor-related protein 5 gene or lifestyle factors in Japanese female workers. *Asia Pacific Journal of Clinical Nutrition*.

[22] Falcón-Ramírez E., Casas-Avila L., Cerda-Flores R. M., Castro-Hernández C., Rubio-Lightbourn J., Velázquez-Cruz R., Diez-G P., Peñaloza-Espinosa R., Valdés-Flores M. (2013). Association of LRP5 haplotypes with osteoporosis in Mexican women. *Molecular Biology Reports*.

[23] Genant H. K., Cooper C., Poor G., Reid I., Ehrlich G., Kanis J., Nordin B. E. C., Barrett-Connor E., Black D., Bonjour J.-P., Dawson-Hughes B., Delmas P. D., Dequeker J., Eis S. R., Gennari C., Johnell O., Johnston C.C. J., Lau E. M. C., Liberman U. A., Lindsay R., Martin T. J., Masri B., Mautalen C. A., Meunier P. J., Miller P. D., Mithal A., Morii H., Papapoulos S., Woolf A., Yu W., Khaltaev N. (1999). Interim report and recommendations of the World Health Organization Task-Force for Osteoporosis. *Osteoporosis International*.

[24] Nakamura T. (2007). Absolute risk for fracture and WHO guideline: fracture risk assessments recommended by World Health Organization and Japanese guidelines for prevention and treatment of osteoporosis 2006. *Clinical calcium*.

[25] Stang A. (2010). Critical evaluation of the Newcastle-Ottawa scale for the assessment of the quality of nonrandomized studies in meta-analyses. *European Journal of Epidemiology*.

[26] Zintzaras E., Ioannidis J. P. A. (2005). HEGESMA: genome search meta-analysis and heterogeneity testing. *Bioinformatics*.

[27] Zintzaras E., Ioannidis J. P. A. (2005). Heterogeneity testing in meta-analysis of genome searches. *Genetic Epidemiology*.

[28] Higgins J. P., Thompson S. G. (2002). Quantifying heterogeneity in a meta-analysis. *Statistics in Medicine*.

[29] Song F., Gilbody S. (1998). Bias in meta-analysis detected by a simple, graphical test: increase in studies of publication bias coincided with increasing use of meta-analysis. *British Medical Journal*.

[30] Peters J. L., Sutton A. J., Jones D. R., Abrams K. R., Rushton L. (2006). Comparison of two methods to detect publication bias in meta-analysis. *Journal of the American Medical Association*.

[31] Ferrari S. L., Deutsch S., Baudoin C., Cohen-Solal M., Ostertag A., Antonarakis S. E., Rizzoli R., De Vernejoul M. C. (2005). LRP5 gene polymorphisms and idiopathic osteoporosis in men. *Bone*.

[32] Mizuguchi T., Furuta I., Watanabe Y., Tsukamoto K., Tomita H., Tsujihata M., Ohta T., Kishino T., Matsumoto N., Minakami H., Niikawa N., Yoshiura K.-I. (2004). LRP5, low-density-lipoprotein-receptor-related protein 5, is a determinant for bone mineral density. *Journal of Human Genetics*.

[33] Xuan M., Wang Y., Wang W., Yang J., Li Y., Zhang X. (2014). Association of LRP5 gene polymorphism with type 2 diabetes mellitus and osteoporosis in postmenopausal women. *International Journal of Clinical and Experimental Medicine*.

[34] Liu J. M., Zhang M. J., Zhao L., Cui B., Li Z. B., Zhao H. Y., Sun L. H., Tao B., Li M., Ning G. (2010). Analysis of recently identified osteoporosis susceptibility genes in Han Chinese women. *Journal of Clinical Endocrinology and Metabolism*.

[35] Hartikka H., Mäkitie O., Männikkö M., Doria A. S., Daneman A., Cole W. G., Ala-Kokko L., Sochett E. B. (2005). Heterozygous mutations in the LDL receptor-related protein 5 (LRP5) gene are associated with primary osteoporosis in children. *Journal of Bone and Mineral Research*.

[36] Strickland D. K., Gonias S. L., Argraves W. S. (2002). Diverse roles for the LDL receptor family. *Trends in Endocrinology and Metabolism*.

[37] Koay M. A., Brown M. A. (2005). Genetic disorders of the LRP5-Wnt signalling pathway affecting the skeleton. *Trends in Molecular Medicine*.

[38] Gong Y., Slee R. B., Fukai N. (2001). LDL receptor-related protein 5 (LRP5) affects bone accrual and eye development. *Cell*.

[39] Bollerslev J., Wilson S. G., Dick I. M., Islam F. M. A., Ueland T., Palmer L., Devine A., Prince R. L. (2005). LRP5 gene polymorphisms predict bone mass and incident fractures in elderly Australian women. *Bone*.

[40] Korvala J., Jüppner H., Mäkitie O., Sochett E., Schnabel D., Mora S., Bartels C. F., Warman M. L., Deraska D., Cole W. G., Hartikka H., Ala-Kokko L., Männikkö M. (2012). Mutations in LRP5 cause primary osteoporosis without features of OI by reducing Wnt signaling activity. *BMC Medical Genetics*.

